# Quantifying Light Harshness: Method Automation and Influence of Photographic Light Modifiers

**DOI:** 10.3390/jimaging12040148

**Published:** 2026-03-27

**Authors:** Veronika Štampfl, Jure Ahtik

**Affiliations:** Chair of Information and Graphic Arts Technology, Department of Textiles, Graphic Arts and Design, Faculty of Natural Sciences and Engineering, University of Ljubljana, 1000 Ljubljana, Slovenia; jure.ahtik@ntf.uni-lj.si

**Keywords:** light harshness, shadow analysis, image processing, lighting evaluation, photographic modifiers

## Abstract

Accurate assessment of light properties is essential and is measured with photometric and colorimetric standardized methods. However, the spatial characteristic of light—harshness—remains difficult to quantify. Building on the authors’ previous work, this study presented a fully automated method for determining light source harshness based on image analysis of cast shadows in a standardized environment. The improved method eliminated the need for manual shadow segmentation by introducing algorithmic noise removal and adaptive smoothing of shadow data. The method was applied to 180 test images comprising 30 combinations of photographic light-shaping attachments (e.g., softboxes, beauty dishes, and snoots) across two light sources (halogen and xenon) and three intensity levels. The results showed that the method was capable of detecting subtle differences in shadow properties and confirmed the influence of geometry, material, and orientation of the light modifiers on harshness. In addition, the results provided quantitative insight into the influence of photographic light modifiers on the original light.

## 1. Introduction

Artificial lighting has a profound impact on various fields such as photography, cinematography, architecture, industrial design, and human-centered visual systems. These fields often rely on precise and predictable lighting conditions. Lighting quality is traditionally quantified by photometric and colorimetric parameters, such as luminous intensity, correlated color temperature (CCT), and color rendering index (CRI), which are internationally standardized [1,2]. Although these parameters are of fundamental importance, they do not capture the other perceptual qualities, such as harshness (also perceived as softness of light or shadow edges) or visual comfort of the lighting [3].

In our previous research [4], we developed a method that quantitatively describes the harshness of a tested light source. However, the method was not fully automated and was developed as a proof of concept for a sparse selection of light sources with different properties. Therefore, the objective of this research is to fully automate the originally proposed method and apply it to a more extensive and diverse set of lighting conditions, i.e., a selection of light-shaping attachments, causing differences in light harshness of the light source’s beam.

### 1.1. Light Harshness in Photographic Lighting

Light harshness refers to the spatial intensity distribution and edge definition of the shadows cast by an illuminated object. Softboxes, beauty dishes, honeycomb grids, reflectors, diffusion panels, and other light-shaping attachments are commonly used to alter harshness by diffusing or directing light. The design, materials, and geometry of these attachments critically shape the light beam profile and, in addition, lead to different shadows, which can influence the viewer’s perception and focus [3,5].

Although this concept is central to lighting design, the evaluation of their performance remains largely qualitative [5]. Inconsistencies in the setup, the geometry of the scene, and subjective assessments make comparative studies challenging. Therefore, a reproducible, quantitative, and scalable method for evaluating light harshness is essential.

### 1.2. Shadow Analysis and Shadow Removal

Shadows provide a reliable visual clue for analyzing the directional and spatial properties of light sources. The structure of cast shadows—specifically the geometry and intensity gradient between umbra and penumbra—has been used to draw conclusions about light direction, softness, and environment [5,6]. In particular, Basri and Jacobs [7] and Sato et al. [8] have shown that geometric shadow analysis can reveal physical properties of illumination sources.

Building on this, more recent methods in computer vision have used segmentation and learning-based techniques to detect shadows, especially in uncontrolled outdoor environments. Dong et al. [9], Khan et al. [10], and Chen et al. [11] developed algorithms using edge, intensity, and learning models for shadow detection. Similarly, Hu et al. [12] proposed direction-aware spatial context features to improve shadow removal.

Studies by Yasutomi et al. [13] and Imai et al. [14] address shadow estimation in ultrasound and hyperspectral UAV (drone) imagery, suggesting a broader relevance and transferability of methods for quantifying shadows in different domains. In addition, Reddy and Harikiran [15], Zhang and Kong [16], and Zhang et al. [17] propose robust models for angle detection, recurrent attention, and multiscale decomposition in the shadow context, reflecting the current trajectory of research in combining physical shadow features with AI-driven interpretation.

Advanced techniques for image shadow removal, such as the utilization of conditional generative adversarial networks (CGAN) for shadow detection followed by its removal, have shown promise in both natural [17,18] and technical [19] imaging domains. For example, GAN-based models were used for cast shadow removal by Xu et al. [20] and Acharya and Thapa [21]. Inoue and Yamasaki [22] utilized synthetic shadows for deep learning-based shadow detection and removal. While these advances improve visual clarity, they are not primarily focused on quantifying shadow structure and light harshness.

### 1.3. Determining Light Harshness

In our previous research [4], we introduced a novel method that uses binary thresholding for shadow segmentation, which allows us to distinguish umbral and penumbral regions in cast shadows, leading to shadow harshness estimation and, with that, also light harshness estimation since the shadow is the consequence of the light.

The method assumes a standardized setup for capturing an image of a shadowed surface that is also predefined, as well as the occluder casting the shadow. The method was tested for LED, halogen, and xenon light sources, while the beam was additionally modified with a light-shaping attachment to spread the light. The research showed that the method is most efficient when the contrast of the shadow to the background is higher, leading to a more precise harshness estimation.

**Figure 1:**
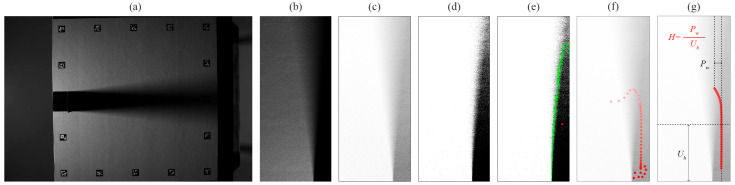
Visual overview of the method: (**a**) input image, (**b**) aligned and cropped test image, (**c**) extracted shadow, (**d**) example of a thresholded image (at threshold 128), (**e**) detected contour (green) and its centroid (red), (**f**) sequence of centroids for all 256 thresholded images, and (**g**) final shadow gradient with the breaking point defined at the intersection of the two dotted lines, dividing the shadow into umbra and penumbra, leading to final harshness calculation.

Key steps of the method are shown in Figure 1. Firstly, the input image of the shadowed surface is aligned to the reference image and cropped to the area of interest. Secondly, the shadow is extracted with background removal, and thirdly, the remaining shadow image is thresholded at every threshold, resulting in 256 binary images. Then, the centroid of the binary shape is calculated and added to the data stack to form a dataset with 256 data points outlining the shadow gradient, as shown in Figure 1f. Noise in the dataset is then removed with visual analysis, while the breaking point from umbra to penumbra is mathematically defined. The final harshness value H is based on the ratio of penumbra width $P_{w}$ to umbra height $U_{h}$.

This approach was applied to different types of light sources with and without photographic modifiers and established harshness as a quantifiable metric associated with shadow intensity ratios. Although promising, the technique required manual noise removal steps followed by visual validation and lacked a wide span of test situations.

## 2. Proposed Method Automation

Firstly, we present the algorithmic overview of the original method, which has been published as a novel method providing a numerical estimation of shadow harshness and, with that, the tested light source harshness as well [4]. Secondly, we present the proposed method extension, which includes additional steps to improve the original method and enable full method automation, eliminating visual analysis steps to determine data noise or shadow boundaries.

### 2.1. Original Method Overview

The original method for light harshness determination can be divided into four sections: image registration, shadow detection, shadow segmentation, and final harshness determination. In this subsection, we briefly describe each segment and highlight the sections of the method that have been improved throughout this study.

The required input data is an image of a shadowed region and a corresponding image of the identical area that was not shaded but still illuminated with the same tested light source. These test images should be taken in a special laboratory setup proposed as a standardized test environment for harshness determination, allowing full comparability regardless of the tested light. The reference image required for image registration is also predefined.

With image registration, the comparability of the tested images is ensured. This is achieved by registering the test images on the reference image. The latter consists of ArUco markers placed identically to those on the projection plane, so that there are enough reference points to determine the overlap of otherwise visually empty areas. First, the test photos are converted from RAW to JPG format and then from RGB color space to grayscale. ArUco markers are recognized on both the reference and test image, which allows the calculation of a homography matrix through which the test image is registered to the reference. The aligned images are then cropped to a specifically predefined area, splitting the shadow in half along its vertical axis and removing areas without shadows. The final cropped images are saved in 8-bit JPG format.

Shadow detection requires two aligned and cropped images. The pair consists of a shadowed image, $I_{SB}$, and a non-shadowed image, $I_{B}$, i.e., a background image. These are then subtracted and inverted to obtain the positive form of the shadow, image I. To extract the information of the shadow shape at different brightness levels, an inverse binary threshold is applied to this image at each of the 256 thresholds $Y_{thr}$ (varying from 0 to 255 in steps of 1, here denoted as i), resulting in 256 binary images. In each of these images, the centroid of the most prominent contour representing the shadow shape is calculated. This results in a set of 256 centroid coordinates $\left(\bar{x},\bar{y}\right)$ that outline the gradient of the analyzed shadow.

However, in some thresholded images, the shape of the shadow might not be identifiable, so the centroids cannot be calculated. In these cases, the centroid is set to (0, 0). In other cases, the gradients may not appear constant in their progression and show clear noise during visual inspection, either repetitive or random. This noise is removed manually, while the outliers are determined by visual analysis. Only then can the segmentation of the shadow be done.

For shadow segmentation, the threshold values where the shadow starts ($S_{s}$), and ends ($S_{e}$) are detected in the first and last values where coordinates are not (0, 0). This range is defined as the shadow range $S_{r}$, which is then smoothed using a Savitzky–Golay filter [23] with an adaptive window length. The curve $\tilde{y}=g(\tilde{x})$ is then formed through the filtered datasets, representing the shadow shape. To determine where the umbra ends and the penumbra begins, a transition point $S_{u/p}$ is defined with the following equation

(1) $$S_{u/p}=\left\{\begin{array}{l} \max_{i}\left(g^{\prime }\left({\tilde{x}}_{i}\right)\right)\;\;\;\;\;\;,\;\;\mathrm{if}\;\;{\tilde{x}}_{j}<{\tilde{x}}_{i}\;,\;\forall \;j<i\\ \max_{i=1,\ldots ,i_{\max}}\left(g^{\prime }\left({\tilde{x}}_{i}\right)\right),\;\;\mathrm{otherwise} \end{array}\right.,$$

where $g\prime (\tilde{x})$ is the derivative of the fitted curve, and $i_{max}$ is the positional argument of its maximum value.

When the transition from the umbra to the penumbra is defined, umbra height $U_{h}$ and penumbra width $P_{w}$ can be determined using the following equations

(2) $$U_{h}=1-{g(\tilde{x})}_{S_{u/p}},$$

and

(3) $$P_{w}=\left|{\tilde{x}}_{S_{u/p}}-{\tilde{x}}_{S_{e}}\right|.$$

These two values make it possible to calculate harshness H:

(4) $$H=\frac{P_{w}}{U_{h}}.$$

### 2.2. Proposed Extension for Method Automation

Although the original method provides quantitative results that correlate with qualitative assessments of shadow harshness—and consequently the light and light source harshness [4]—it is not fully automated. Its main limitation lies in the manual removal of noise, which is mainly caused by pixel-based image analysis techniques. In this study, a larger input dataset allowed the method to be fully automated with a method extension.

We followed the original method with a four-section structure, not changing the processes within image registration, shadow detection, and final harshness evaluation. Improvements are proposed in the shadow segmentation stage, where both repetitive and random noise are algorithmically removed, and manual intervention is no longer required. The detection of the transition point $S_{u/p}$ has been updated to be applicable for a wider range of light sources.

First, we dealt with the repetitive noise visible in the lowest and highest threshold values $Y_{thr}$—in the beginnings and ends of the centroid coordinate sets of $\left(\bar{x},\bar{y}\right)$. Therefore, we had to determine the beginning of the shadow $S_{s}$ and the end of the shadow $S_{e}$ algorithmically. Instead of determining the noise limits visually, we first calculated the derivatives

(5) $${\bar{x}}^{\prime }=\frac{\Delta \bar{x}}{\Delta Y_{thr}},$$

and

(6) $${\bar{y}}^{\prime }=\frac{\Delta \bar{y}}{\Delta Y_{thr}},$$

where $\Delta \bar{x}$ and $\Delta \bar{y}$ are the differences between the two sequence coordinates of $\bar{x}$ and $\bar{y}$, and $\Delta Y_{thr}$ is the difference between two sequence thresholds, i.e., 1. Then, we determined a series of four breakpoints that enabled the detection of $S_{e}$. With ${\bar{y}}_{max}$, we determined the positional argument of the maximum value in the set of $\bar{y}$ coordinates and shifted this value by 5 to avoid the detection of false maxima that could be due to random noise:

(7) $${\bar{y}}_{max}=\underset{i=0,\ldots ,255}{\mathrm{arg max}}\left({\bar{y}}_{i}\right)+5.$$

Then we have determined ${\bar{y}\prime }_{last\_pos}$, the positional argument of the last positive value in the set of $\bar{y}$ coordinates:

(8) $${\bar{y}\prime }_{last\_pos}=max\;\left\{\;i\;|\;{\bar{y}\prime }_{i}\geq 0,\;\;{\bar{y}}_{max}\leq i\leq 255\right\}.$$

This allowed setting the limits to determine ${\bar{x}}_{min}$ and ${\bar{y}}_{min}$, both positional arguments in their own sets of coordinates $\bar{x}$ and $\bar{y}$:

(9) $${\bar{x}}_{min}=\underset{{\bar{y}}_{\max}\;<\;i\;\leq \;255}{\mathrm{arg min}}\left({\bar{x}}_{i}\right),$$

and

(10) $${\bar{y}}_{min}=\underset{{\bar{y}}_{\max}\;\leq \;i\;<{\bar{y}\prime }_{\mathrm{last}\_\mathrm{pos}}}{\mathrm{arg min}}\left({\bar{y}}_{i}\right).$$

These were then applied to the algorithmic determination of the shadow end $S_{e}$:

(11) $$S_{e}=\left\{\begin{array}{cc} {\bar{y}}_{min} & ,\;if\;{\bar{y}}_{min}<{\bar{x}}_{min}\\ {\bar{x}}_{min}-1 & ,otherwise \end{array}\right..$$

To find the shadow start $S_{s}$, we first found the positional argument of the last negative element in the set of $\bar{y}$ coordinates

(12) $${\bar{y}\prime }_{last\_neg}=max\;\left\{\;i\;|\;{\bar{y}\prime }_{i}<0,\;\;\;\;i<S_{e}\right\},$$

and used this value to find the longest sequence of negative values in the dataset ${\bar{y}}^{\prime }[:{\bar{y}\prime }_{last\_neg}]$. We determined $S_{s}$ in the first element of the longest sequence.

To maintain the comparability of the datasets, we did not delete the data points outside the shadow start and shadow end ranges but replaced them with (0,0) to retain their length. We then combined the data arrays for the same light source combination, which differed only in intensity, and rearranged the data points—this time according to the descending $\bar{y}$ value rather than the polar angle, as the original method suggested. If one of the coordinates was duplicated, we kept only one.

To remove the random noise, we calculated the Euclidean distance d(A,B) for each two sequence coordinates $A({\bar{x}}_{i},{\bar{y}}_{i})$ and $B({\bar{x}}_{i+1},{\bar{y}}_{i+1})$ in a dataset:

(13) $$d(A,B)=\sqrt{{\left({\bar{x}}_{i+1}-{\bar{x}}_{i}\right)}^{2}+{\left({\bar{y}}_{i+1}-{\bar{y}}_{i}\right)}^{2}}.$$

If the distance was greater than 0.02, we defined the coordinate $\left({\bar{x}}_{i},{\bar{y}}_{i}\right)$ as noise and removed it from the dataset.

We then proceeded to smooth the datasets, applying Gaussian filtering instead of the Savitzky–Golay filter due to the more uniform nature of the data. The smoothing parameter σ was adjusted for each dataset using the following equation

(14) $$\sigma =\min\left(\frac{L*0.1}{6},2\right)=min\left(\frac{L}{60},2\right),$$

where L is the length of the dataset. This ensured an effective smoothing window covering approximately 10% of the length of the dataset (following the commonly used 6σ rule for the size of the Gaussian kernel [24]), while the maximum smoothing is limited to σ=2 to prevent over-smoothing. As the original method proposes, we continued with the spline fitting and fitted a curve $\tilde{y}=g(\tilde{x})$ through consecutive $\left({\bar{x}}_{i},{\bar{y}}_{i}\right)$ coordinates.

To determine the transition point $S_{u/p}$, we have again defined two breakpoints—${\tilde{x}}_{max}$ and ${\tilde{y}\prime }_{max}$:

(15) $${\tilde{x}}_{max}=\underset{i=0,\ldots ,\left|\tilde{x}\right|}{\mathrm{arg max}}\left({\tilde{x}}_{i}\right)$$

and

(16) $${\tilde{y}\prime }_{max}=\underset{i=0,\ldots ,\left|\tilde{x}\right|}{\mathrm{arg max}}\left(g^{\prime }\left({\tilde{x}}_{i}\right)\right)\;,$$

where $g^{\prime }\left({\tilde{x}}_{i}\right)$ is the derivative of $g\left({\tilde{x}}_{i}\right)$. Then we could set up conditions for $S_{u/p}$ according to

(17) $$S_{u/p}=\left\{\begin{array}{cc} 0 & ,\;if\;{\tilde{x}}_{max}=0\;and\;\forall \;i,\;\;{\tilde{x}}_{i}>{\tilde{x}}_{i+1}\\ {\tilde{x}}_{max} & ,if\;{\tilde{x}}_{max}>{\tilde{y}\prime }_{max}\\ {\tilde{y}\prime }_{max} & ,otherwise \end{array}\right.\;.$$

Umbra height $U_{h}$ and the penumbra width $P_{w}$, followed by the harshness H, were then calculated according to Equations (2)–(4), as predicted by the original method.

## 3. The Experiment: Influence of Light Modifiers on Light Harshness

We tested a series of 30 different combinations of the original light source and an attached light-shaping attachment (modifier). This allowed a large enough dataset for full automation of the method and a more accurate determination of the harshness *H* for a wider range of light source combinations.

### 3.1. Tested Light-Shaping Attachments and Light Sources

**Figure 2:**
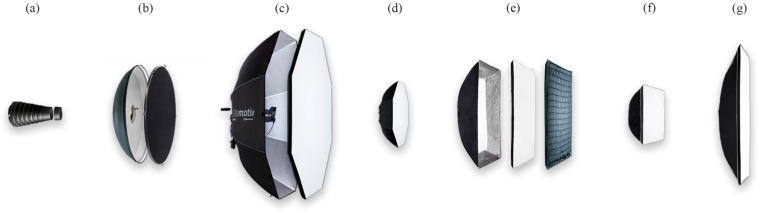
Tested light-shaping attachments: (**a**) snoot with grid; (**b**) beauty dish reflector with a silver deflector and a grid; (**c**) large indirect softbox—octabox; (**d**) small direct softbox—octabox; (**e**) large square softbox—squarebox with inner liner, outer cover, and grid; (**f**) small square softbox; and (**g**) stripbox in vertical position.

We have decided on three general types of photographic light-shaping attachments: snoot, beauty dish, and softbox. The three types are shown in Figure 2, while the softbox had three variants that differed in shape and size: stripbox, square softbox (squarebox), and an octabox (Figure 2c–g). Each of the five attachments was tested in several variations, depending on what it offered: with and without grids to direct the light, with and without deflectors, and/or with and without inner liner and outer cover to soften/spread the light. The overview of all 30 combinations is shown in Figure 2 and Table 1.

The first type was a beauty dish reflector in seven combinations, each including the main *Elinchrom Softlite Silver Beauty Dish Reflector* (Elinchrom, New York, USA; applies to all Elinchrom products in this study) with a diameter of 70 cm (label *BD_R*). The combinations differed in the characteristics of the added deflector from the *Elinchrom Deflector Kit* (gold—*DG*, silver—*DS*, and white—*DW*), while we tested them with and without the added grid (label *G*), *Elinchrom Softlite Beauty Dish Grid*.

The second type of light shapers were softboxes, which differed in size, shape, and manufacturer. The largest of these was the octabox *Elinchrom Indirect Litemotiv Octa Softbox* (190 cm diameter), which reflects the light indirectly. A smaller version with the same shape was the *Elinchrom Portalite Octa Softbox* (56 cm diameter), but with direct lighting. Both were tested in two combinations: the first consisted only of the outer main part of the modifier—the box (label *B—Elin.*), while the second included a white outer diffuser—cover (label *C—Elin.*).

Two other square softboxes—squareboxes—were tested, namely the *Elinchrom Portalite Softbox* (66 cm side length) and the *Elinchrom Rotalux Square Softbox* (100 cm side length). Both were tested only with the reflective box and in combination with the attached cover. The larger modifier allowed the addition of a grid and an inner diffuser—liner (label *L—Elin.*), which we tested in all available combinations. A rectangular softbox—stripbox was also tested in two orientations: horizontal and vertical. We tested the *Quadralite Softbox* (Quadralite, Krakow, Poland) with dimensions of 30 × 120 cm in four combinations of box, liner, and cover (labels *B—Quad.*, *L—Quad.*, and *C—Quad.*).

The third type of light modifier was the *Elinchrom Snoot Reflector* cone, which we tested in two combinations—with and without an additional grid with 15° angled blades.

**Figure 3:**
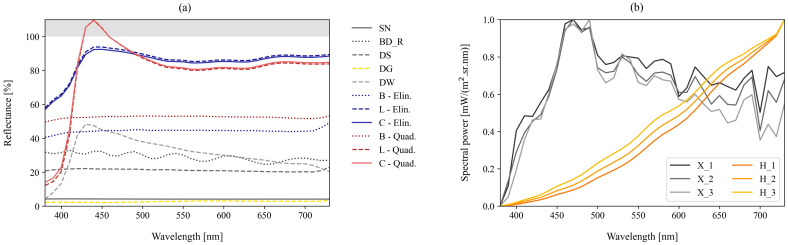
(**a**) Reflectance spectra of the materials of the overall surfaces of the tested light-shaping attachments and (**b**) normalized emission spectra of the tested light sources at three intensity levels.

In order to better understand the material properties, we measured the reflectance spectrum of the materials of the overall surfaces of the tested light-shaping attachments. Due to the limitation of small and inaccessible surfaces, we could not determine the surface properties of the grids. These properties were measured using an *X-Rite i1 Pro* 2 spectrophotometer (X-Rite, Grand Rapids, MI, USA) and *Argyll CMS* software (version 1.3) and are shown in Figure 3a.

To determine whether the light modifiers interact differently with different light sources, we included two types of light sources in the study: a *Kaiser Studiolight H* light source (Kaiser Fototechnik, Buchen, Germany) continuous with an *Osram 64,575* halogen lamp (23 V, 1000 W; Osram, Munich, Germany) and an *Elinchrom Pro HD 500* flash unit (Elinchrom, Renens, Switzerland) with an *Elinchrom ELC Pro HD* xenon flash tube (Elinchrom, Renens, Switzerland). These two types of light sources are the most commonly used types in photography, while the LED was not included because it was not possible to attach the identical light-shaping attachments to the base of the light source.

We tested the light sources at three intensity levels to eliminate the effect of inconsistent light source characteristics at different wattages. Halogen was tested at 57 lx, 99 lx, and 225 lx, and xenon was tested at 3268 lx, 6822 lx, and 12,786 lx. We measured the emission spectra for each sample using an identical spectrophotometer setup as for the surface measurements of the modifiers, normalizing the emission spectra to the range 0 to 1, as shown in Figure 3b. The correlated color temperature (CCT) ranged from 2425 K to 2757 K for halogen and from 5893 K to 6012 K for xenon, generally increasing with light intensity.

### 3.2. Test Environment and Test Images

While the original study used a darkroom as the test environment [4], we had to use a larger room due to the size of the light modifiers tested. We placed the entire test setup in a photo studio with a black background, which proved to have a comparable effect on the observed scene as the originally used darkroom when the tested light source combination is directed at the background [25].

**Figure 4:**
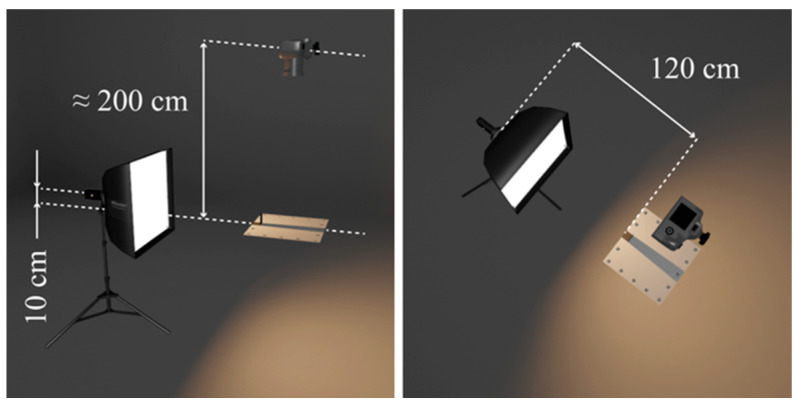
Render of a test scene with indicated measurements.

For shadow generation, we used the proposed standardized test scene, which consists of a 50 cm square gray projection surface with 16 ArUco markers distributed along the edges and a 6 × 8 cm L-shaped aluminum profile for casting shadows. A 3D representation of the scene can be seen in Figure 4, along with the distances predicted in the original study.

We captured a flat image of the projection plane with and without a shadow area (with and without an occluder) to form a test pair for each of the 180 light source combinations (30 modifiers and 2 light sources at 3 intensities). We used a *Nikon D850* camera with a *Nikkor 50 mm 1.4G* lens (Nikon, Tokyo, Japan), with no variation in camera settings for a given light source. All images were captured in RAW format to minimize data loss.

### 3.3. Calculating the Effect of Light-Shaping Attachments

Since the study includes light sources in combination with different light-shaping attachments, we were able to determine the difference in harshness $H_{M}$ caused by the modifier. To determine this difference, we calculated the harshness H for every tested combination of the light source and a light modifier, and then calculate $H_{M}$ with equation

(18) $$H_{M}=H_{LM}-H_{L},$$

where $H_{LM}$ is the harshness of the light source in combination with the modifier, and $H_{L}$ is the harshness produced only by the light source.

## 4. Results and Discussion

This research focuses on improving the original method of detecting the shadow shape from a standardized image with the intent of determining the light harshness, as well as the characterization of a wider range of lighting conditions. First, we comment on the success of the proposed method improvements, and secondly, we analyze the differences in harshness caused by applying different light-shaping attachments to the original light source. An overview of the main results is presented in the third subsection, followed by a critical assessment of the proposed method automation, results, and method applicability.

### 4.1. Method Automation

**Figure 5:**
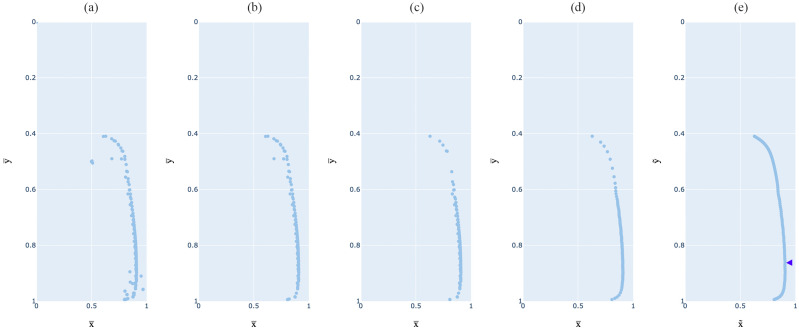
Result overview for one of the test combinations in five stages of the improved method: (**a**) original dataset—centroids, (**b**) algorithmically removed repetitive noise, (**c**) algorithmically removed random noise, (**d**) applied Gaussian filtering, and (**e**) fitted curve $\tilde{y}=g(\tilde{x})$ with marked transition point $S_{u/p}$ (blue arrow).

To achieve full automation of the method, we developed an algorithm to automatically remove noise in the datasets. The first step involved the removal of repetitive noise seen at the beginning and end of each dataset (i.e., at the darkest and brightest parts of the image) and most likely caused by the removal of the background, the unevenness of the projection plane surface, or the dynamic range of the camera, each of them possibly leading to noise in the analyzed images. This noise can be seen in the input dataset in Figure 5a, while in Figure 5b it is removed.

**Figure 6:**
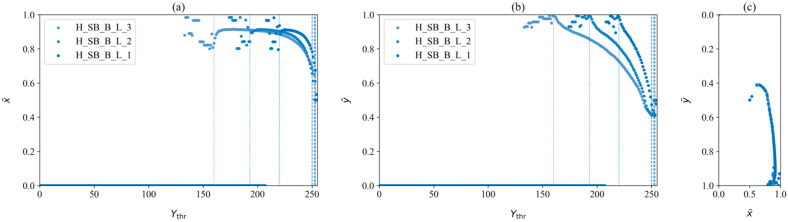
(**a**) $\bar{x}$ coordinates, (**b**) $\bar{y}$ coordinates, and (**c**) both coordinates of the centroids for H_SB_B_L in three light source intensity levels, with the dotted line marking the beginning of the shadow $S_{s}$, and the dashed line is the end of the shadow $S_{e}$.

The success rate of noise removal was visually monitored to achieve consistent trends in the dataset. While the visualization from Figure 5a,b could allow for errors in visually understanding the data due to the dense scatter of coordinates and their order not being visible, another visualization was used to monitor the noise removal process, as shown in Figure 6. It shows the $\bar{x}$ and $\bar{y}$ coordinates separately as a function of the threshold $Y_{thr}$, with clear, consistent trends plotted in the range from the shadow start $S_{s}$ to shadow end $S_{e}$. While this approach proved to be efficient in determining $S_{s}$ and $S_{e}$, there were still coordinates that needed to be filtered out, either near these two points or randomly within the dataset. These coordinates were removed in the next step as they were detected as random noise.

The random noise represents the noise that could not have been explained by any shadow property. We successfully defined it with the Euclidean distance between two consecutive points. A comparison of Figure 5b,c shows how the random noise was correctly defined and removed, with two coordinates at approximately $\bar{y}=0.5$ being visually perceived as outliers. However, in some datasets, this approach led to incorrect detection of noise and, with that, removal of these points, making the dataset sparser. This can also be seen in Figure 5c at $\bar{y}=0.5$.

Nevertheless, this did not affect the quality of the data in any of the 180 datasets, as the process was followed by Gaussian filtering that interpolated the datasets while still following the data trend, as can be seen in Figure 5d. This allowed the final fitting of the curve $\tilde{y}=g(\tilde{x})$ without a raised error (Figure 5e).

**Figure 7:**
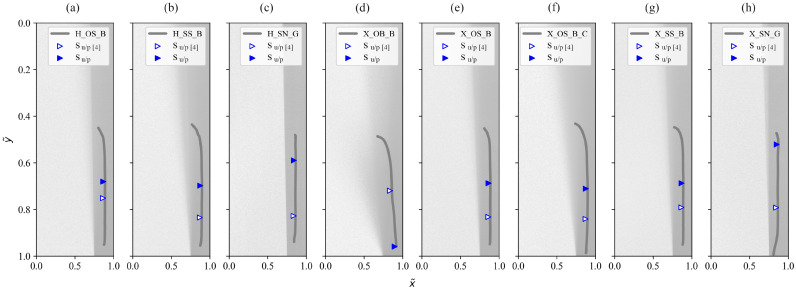
Transition point $S_{u/p}$ determined using the equation from the original method [4] and the updated approach from this study for test combinations: (**a**) H_OS_B; (**b**) H_SS_B; (**c**) H_SN_G; (**d**) X_OB_B; (**e**) X_OS_B; (**f**) X_OS_B_C; (**g**) X_SS_B; (**h**) X_SN_G.

While the original approach to determining the transition point from the umbra to penumbra $S_{u/p}$ was applicable to the limited dataset from the original study [4], weaknesses arose when applied to a wider range of light properties. This study provided shadow data for 60 different combinations of light sources and light-shaping attachments, which allowed the algorithm to be improved. Figure 7 shows the shadow gradients for eight combinations, all of which gave a different result when the updated algorithm was used. In all but one case, the $S_{u/p}$ was detected at a lower $\tilde{y}$ value (visually higher on the plot), resulting in a higher umbra height value $U_{h}$ and consequently, a different harshness value H. Nevertheless, the $U_{h}$ is balanced with the penumbra width $P_{w}$, so the differences in the results for these three values are not linear. The only combination with the opposite result is X_OB_B, which is also the only combination that satisfies the first condition from (17). However, this condition is essential, as otherwise $S_{u/p}$ would not be detected until much later in the dataset, resulting in harshness value H that does not match the visually perceived harshness and would indicate that two light-shaping attachments behave the same, while in reality they differ in size, shape, and type of materials, and the result cannot be similar.

To determine whether the conditions applied in (17) are appropriate, we first visually compared the shadow gradient formed by $\tilde{y}=g(\tilde{x})$ with the underlying shadow image (as in Figure 7), followed by a comparison of the $S_{u/p}$ values and their effect on harshness H. In addition, we compared the results for test combinations that differed only in the type of light source (e.g., H_OS_B and X_OS_B), as we know empirically that differences in harshness can occur, but only to a small extent. These comparisons allowed us to confirm the applicability of set algorithmic conditions, since the results now matched otherwise visually perceived shadow properties. The most obvious example is the comparison of $S_{u/p}$ for H_OS_B and X_OB_B in Figure 7a,d, where the value was similar according to the original method, but visually, the shadow harshness is much softer for X_OB_B. This was also shown by the shadow gradient but was not reflected by $S_{u/p}$. According to the proposed method extension, these two values differ significantly and correlate with visually perceived harshness.

### 4.2. Effect of Light-Shaping Attachments on Original Light

In addition to the automation of the method, this study also focuses on the characterization of the specific light-shaping attachments on the original light, as the harshness value H in combination with our empirical knowledge was the general approach to evaluate the success rate of the method improvement.

**Figure 8:**
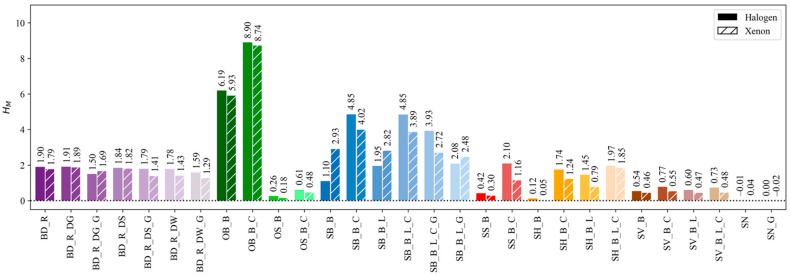
Differences in light harshness $H_{M}$ as a result of light modifiers applied to a halogen and xenon light source.

Two light sources were characterized—halogen and xenon—each without and with one of the 29 light modifiers in place, resulting in 60 test combinations. While the harshness level of the halogen light source is H=0.01, and for xenon it is H=0.07, the influence of each modifier was determined as $H_{M}$, where a positive value indicates softening of the original light, i.e., a scattering of the light beam. The results are shown in Figure 8.

The beauty dish modifier (BD) shows consistent values in harshness change, regardless of the modifier combination used. The average values for the seven test combinations are 1.76 and 1.62 for halogen and xenon light sources, with one of the smallest standard deviations (0.16 and 0.24, respectively). This indicates that the deflectors, although they differ in their optical properties, do not drastically affect the harshness of the original light, but they provide similar results, even when compared with a sole reflector and an additional grid.

The following two combinations for the indirect octabox (OB), which have the largest diameter of the combinations tested, result in the highest harshness change, averaging 7.54 and 7.33 for halogen and xenon, with the largest standard deviation of 1.91 and 1.99, respectively. This indicates that the applied cover softens the light reflected from the outer box of the modifier to a high degree and has the greatest impact on the original light. In contrast, the smaller version of the octagonal softbox (OS) with a direct lighting mechanism, but in combination with identical materials, gives one of the lowest $H_{M}$ values, with 0.44 and 0.33 as the average for halogen and xenon (±0.25 and ±0.22, respectively). This shows that not only the material properties of the light-shaping attachment play a decisive role in light modification, but additional thought should be given to their geometry and light distribution mechanism.

If we compare the results for the large square softbox (SB) with six different material combinations, we can see clear differences in the $H_{M}$ values. While the average value for halogen and xenon is almost identical (3.13 and 3.14), the difference in the corresponding standard deviations is larger—1.62 and 0.65—suggesting that the different material components of the light modifiers may interact differently when coupled with different original light. While for most modifier combinations the results for xenon are slightly lower than the results for halogen, here the simplest combination with the outer box (B) shows a higher level of softening with xenon light. This is also seen for all other combinations with a liner (L) and a grid (G), while the additional cover (C) in conjunction with xenon again produces a harsher light. While both L and C serve the same purpose of softening the original light, C does this at a much higher level, especially in combination with xenon. Both L and C in this case are manufactured by Elinchrom and have matching reflectance spectra (Figure 3a), so the color properties of the material and consequential absorption cannot be the reason for this change in results. While we did not collect such data in this study, it is possible that the reason for this difference lies in the structural properties of the materials, as a previous study has shown [26] that the pore size of the materials can affect certain properties of the light beam, since more light is let through. In addition, L and C differ in their attachment and size, as L is attached 3 cm from the edge of the modifier—the box (B)—and is up to 2 cm smaller than the entire opening of B. These characteristics could influence the degree of effect of the modifier combination on the original light modification.

The trend towards lower $H_{M}$ values for xenon can again be observed in the smaller version of the square softbox (SS), which generally makes the light less soft than its larger version, SB. This can be seen through the average values as well, which are 1.26 for halogen and 0.73 for xenon (±1.19 and ±0.61, respectively). Since the materials used are identical and the SB and SS modifiers differ only in size, we can conclude that the size of the modifier plays a crucial role in these differences.

**Figure 9:**
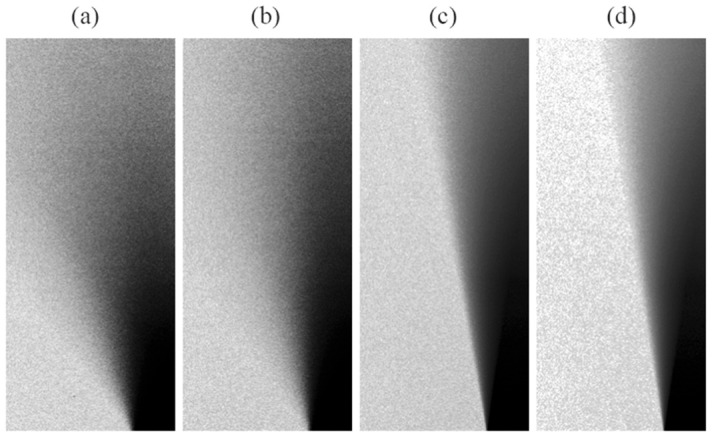
Equalized shadow images for stripboxes in different orientations and coupled with a different light source, all with attached B, L, and C: (**a**) H_SH, (**b**) X_SH, (**c**) H_SV, and (**d**) X_SV.

The narrow softboxes—stripboxes—were tested in two orientations: horizontal and vertical (SH and SV). In the horizontal orientation, the light is softened to a greater extent, as the average values are 1.32 and 0.98 for halogen and xenon, while in the vertical orientation, they are 0.66 and 0.49, respectively. This is due to the geometry of the light shaper, where its horizontal orientation illuminates the projection plane from a wider perspective, resulting in a softer shadow than the vertical orientation, as shown in Figure 9a,b versus Figure 9c,d. While the vertical orientation illuminates the projection plane from a higher viewpoint, which should shorten the shadow produced, especially in the umbra region, and result in a higher harshness value more similar to the SH, this does not happen to the extent that the variation in the orientation does not affect the final $H_{M}$ result. This could be seen as a drawback of the method, as the same modifier could be evaluated with more than one harshness value, or as a feature of the method that provides accurate results depending on the application of modifier orientation. We tend to judge it as the latter, as visual comparison of the images from Figure 9 clearly shows a harsher shadow in vertical orientation, which is consistent with the numerical results from Figure 8.

The results for the SH and SV combination in Figure 8 show a more consistent trend in the variation between samples within each orientation. For SH, the standard deviation is 0.83 for halogen and 0.76 for xenon, and 0.11 and 0.04 for SV, respectively, which may indicate that the material structure of the modifier has less influence on the softening of light when the modifier is narrower or smaller. Nevertheless, both liner and cover (L and C) provide results with a similar trend in light softening. The difference with the previous L and C combinations is the material from which both are made, as they are manufactured by Quadralite and not Elinchrom. While their reflectance spectra peak in the blue region and even reach beyond the 100% mark (Figure 3a), suggesting that they contain optical brighteners, the spectra are consistent, leading us to believe that the material structure (e.g., density and pores) is the reason for the slight difference in the results. This would also support the idea that the combination of L and C produces an even softer light, which is supported by the result for SH_B_L_C.

The last two combinations of light-shaping attachments have a snoot that focuses the light onto a specific surface. Both combinations, with and without the attached grid, show minimal results. The average is 0 for halogen and 0.01 for xenon (±0.01 and ±0.04, respectively), indicating that the light harshness is not affected by the combination of these light-shaping attachments but is merely focused.

### 4.3. Results Overview and Validation

To sum up the extensive results, we can claim that the method provided harshness estimations that are relatable to visually observed properties of the shadows. This led to the conclusion that the noise removal process, which enabled the method automation, was modulated appropriately. The variety of analyzed shadows enabled the definition of the algorithm in multiple steps, where each serves as a safety step in case the previous one was not 100% efficient in defining noise. Consequently, we first defined repetitive noise, followed by random data points that are not conclusive to the data set. Another safety feature that protects the integrity of the datasets is Gaussian filtering, which does not smooth the data to the extent that it would change its properties, but it does make them more uniform, allowing for more precise further analysis.

At the same time, a characterization of 30 light-shaping attachments—light modifiers—was performed. The results differed per modifier depending on the original light properties. This leads us to the conclusion that additional research should be conducted, covering a wider range of light types, especially commonly used LEDs. Since the differences in harshness were mostly linked to the interaction with textile materials, we propose interdisciplinary research, covering also the optical properties of textile fibers. Still, an overview of the results can be summed up according to the type of light modifiers:

- A beauty dish moderately spreads the original light beam, while in combination with the added grid, the spread is slightly lower in harshness value. The use of an additional deflector does not noticeably affect the harshness level.
- Softboxes produce different harshness levels, depending on their size, directivity, orientation, and material properties. Larger and indirect softboxes produce the softest shadows, shown with the highest harshness levels. Smaller softboxes without white overlays do not drastically change the harshness, while denser materials produce higher harshness levels as well. These materials may also interact with original light and deform it differently depending on the light properties.
- Snoot does not significantly change the harshness of the light beam, nor does it in combination with a grid.

While the improved method provided a wide range of quantitative results regarding the effect of light-shaping attachments on the harshness of the original light, we still have concerns regarding the verification of these results. In the original study [4], we already commented that shadow detection cannot be validated with the commonly used BER (balanced error rate), since the latter is based on human perception of the shadow edge, for which it has been shown that it is relative to shadow and background contrast. In addition, statistical analysis as such is not possible, since the maximum harshness value is not defined. Therefore, we propose a study with independent observers who qualitatively compare the shadow images and rate them with a descriptive level of harshness, allowing us to correlate the numerical results more independently.

Such research should be the next step in the evolution of this method, which might already help answer another research hypothesis that emerged when analyzing the results of this study. Namely, we wondered at what level the change can be regarded as an error and can be discarded. In colorimetry, this problem is approached with fixed error values that are not comprehensible to the human eye and sometimes vary according to the observer’s professional experience [27,28]. This type of error threshold could also be applicable in this case, since the aim of the method is to numerically describe a property of light that we otherwise perceive visually.

## 5. Conclusions

The proposed method extension elevated the method’s applicability, since it became fully automated and calls for no manual interventions. This allows it to be used by anyone who follows the experiment, from shadow generation in a standardized environment to image capture and processing.

Despite the opportunities for further research, we judge the improved method as efficient in quantifying the harshness of various light sources, since the full method automation ensured distinction within a wide range of lights produced with light sources in combination with light-shaping attachments commonly used in photographic practice. The results of the harshness study provide the first comprehensive insight into the changes in light harshness that are a consequence of these light modifiers, allowing further development of this area of research.

The method holds utmost value in the field of photography and videography, where creators pick from various light modifiers, while their effect can only be anticipated based on prior experience. In addition, this method estimates the effect of the light modifier on the original light beam quantitatively and allows for product comparison, allowing photographers and videographers to better predict the properties of the light.

## Figures and Tables

**Table 1:** Tested combinations of light-shaping attachments.

Label	Modifier	Combination
0	*none*	*none*
BD_R	reflector; Elinchrom	reflector
BD_R_DG		reflector, gold deflector
BD_R_DS		reflector, silver deflector
BD_R_DW		reflector, white deflector
BD_R_DG_G		reflector, gold deflector, grid
BD_R_DS_G		reflector, silver deflector, grid
BD_R_DW_G		reflector, white deflector, grid
OB_B	indirect octabox, large; Elinchrom	box
OB_B_C		box, cover
OS_B	direct octabox, small; Elinchrom	box
OS_B_C		box, cover
SS_B	squarebox, small; Elinchrom	box
SS_B_C		box, cover
SB_B	squarebox, large; Elinchrom	box
SB_B_L		box, liner
SB_B_L_G		box, liner, grid
SB_B_C		box, cover
SB_B_L_C		box, liner, cover
SB_B_L_C_G		box, liner, cover, grid
SH_B	stripbox, horizontal; Quadralite	box
SH_B_L		box, liner
SH_B_C		box, cover
SH_B_L_C		box, liner, cover
SV_B	stripbox, vertical; Quadralite	box
SV_B_L		box, liner
SV_B_C		box, cover
SV_B_L_C		box, liner, cover
SN	snoot	snoot
SN_G		snoot, grid

## Data Availability

The data presented in this study are openly available in Zenodo at https://doi.org/10.5281/zenodo.18781165.
